# Trajectories and predictors of frailty in older patients undergoing radical prostatectomy: A longitudinal study

**DOI:** 10.1016/j.apjon.2025.100741

**Published:** 2025-06-12

**Authors:** Yongcai Liu, Jieru Zhou, Yijuan Huang, Xin Yao, Xiaoyu Zhang, Jian Cai, Haihong Jiang, Wei Chen, Haiyan Li

**Affiliations:** a Department of Urology, The First Affiliated Hospital of Wenzhou Medical University, Wenzhou, China; b The Second School of Clinical Medical College, Zhejiang Chinese Medical University, Hangzhou, China

**Keywords:** Decision tree, Frailty, Prostate cancer, Trajectory

## Abstract

**Objective:**

To identify the longitudinal heterogeneous trajectories of frailty in older patients with prostate cancer 6 months after radical prostatectomy and to explore the predictors of different trajectories.

**Methods:**

A longitudinal design was conducted, and a total of 248 patients were recruited at the urology department in a tertiary-grade A hospital between June 2024 and March 2025. Data on predictive variables were collected at baseline using General information questionnaire, Geriatric Nutritional Risk Index, Health Literacy Management Scale, Kessler Psychological Distress Scale, and Medical Outcome Study Social Support Survey. Frailty assessments were subsequently performed at four time points using Tilburg Frailty Indicator: hospital discharge, and 1-, 3-, and 6 months postoperatively. Growth Mixture Modeling was employed to examine frailty trajectories over time. Multiple logistic regression and decision tree models were used to explore the predictors of heterogeneous trajectories of frailty.

**Results:**

Three distinct trajectories of frailty were identified in old patients with prostate cancer: frailty rapid improvement group (54.4%), frailty progressive deterioration group (14.5%), and frailty persistent high group (31.1%). Multiple logistic regression analysis identified health literacy, nutritional status, psychological distress, and the presence of two or more comorbidities as independent predictors of frailty trajectories. The decision tree model further highlighted health literacy as the most influential predictor, followed by psychological distress, nutritional status, and social support.

**Conclusions:**

Frailty trajectories in older prostate cancer patients exhibit substantial heterogeneity. These findings provide insights into the predictors of frailty in the process of diagnosis and treatment, and can be used in the clinical identification and monitoring of high-risk patients. Health care providers should develop personalized tailored to these predictive variables to mitigate frailty progression.

## Introduction

Prostate cancer is the most prevalent malignancy among older men globally, with an estimated 1.47 million newly diagnosed cases and 397,430 associated deaths in 2022.^1^ The majority of affected individuals are over the age of 60, making older prostate cancer survivors a large group.^2^^,^^3^ Radical prostatectomy remains a main treatment for prostate cancer, and advances in surgical methods, along with recommendations to base treatment decisions on overall health rather than chronological age, have broadened surgical eligibility.^4^ This has led to an increase in operable cases among elderly patients. Frailty is defined as a clinical condition marked by impaired physiological resilience to stressors, resulting from cumulative declines across multiple systems.^5^ In prostate cancer patients, the combination of diminished physiological reserves of advanced age, and stressors such as cancer diagnosis, surgery, and endocrine therapy can significantly disrupt their baseline health and increase their risk of frailty. A recent cohort study revealed that 33.1% of prostate cancer patients remained in a prolonged frailty state after surgery.^6^ Unfortunately, Frailty in these patients is associated with worse quality of life, inferior overall survival, increased postoperative complications and increased mortality, and other adverse outcomes.^7^^,^^8^ Given its prevalence and significant impact, Early recognition and effective frailty management are essential for improving outcomes among older patients with prostate cancer.

An integral conceptual model of frailty emphasizes its multidimensional and dynamic nature, with previous studies confirming that development trajectories of frailty may improve or deteriorate over time.^9^^,^^10^ Growth mixture modeling (GMM), an advanced statistical method, enable the identification of distinct such subgroups within a population by classifying individuals into a limited number of latent classes.^11^ This analytical approach facilitates a deeper exploration of differences among these subgroups. Existing research highlights the heterogeneity trajectories of frailty in older patients with cancer, underscoring the importance of identifying subgroups with distinct patterns to stratify risks and implement targeted interventions.^12^^,^^13^ Despite these insights, limited research exists on the longitudinal investigations into frailty trajectories among older patients with prostate cancer. This study addresses this gap by applying GMM to identify and analyze heterogeneous frailty trajectories in older patients after radical prostatectomy.

The health ecological model offers a comprehensive framework for understanding the multifactorial nature of frailty.^14^ It encompasses individual traits, personal behaviors, interpersonal networks, living and working conditions, and macro-level factors. Recognizing that frailty development in prostate cancer patients is also shaped by disease-specific characteristics, this study further incorporates the adaptation to the chronic illness framework.^15^ This approach examines how contextual and focal stimuli contribute to frailty development. Therefore, guided by these models, we operationalized demographic characteristics, health literacy, nutritional status, psychological distress, and social support as contextual stimuli, and disease characteristics as focal stimuli, to comprehensively explore predictors of heterogeneous frailty trajectories in prostate cancer survivors after radical prostatectomy.

Previous studies showed that frailty is influenced by a range of factors, including demographic variables such as age, Living condition, education, employment status, and monthly income, as well as disease-related factors like stage of cancer and multicomorbidity.^12^^,^^13^ Moreover, among older patients, malnutrition is a particularly prevalent yet often underappreciated clinical condition, with evidence linking poor nutritional status to an elevated risk of frailty.^16^ Health literacy, defined as the capacity to access, process, and comprehend health-related information, may be another factor of frailty. Limited health literacy can hinder effective communication and access to supportive care, thereby heightening frailty risk.^17^ Similarly, various forms of social support has been identified as a protective factor, buffering against frailty progression in older adults.^18^ However, the unique characteristics of older patients with prostate cancer warrant further investigation to determine whether these findings apply to this population. Notably, psychological distress, encompassing unpleasant emotional experiences such as anxiety and depression, has emerged as a significant correlation with frailty among prostate cancer survivors.^6^ However, this finding is based on cross-sectional studies, which assess variables at a single time point. While such designs are effective for identifying associations, they lack the capacity to determine causal relationships between psychological distress and frailty. These limitations underscored that further investigation is needed to clarify the longitudinal association between these variables and frailty trajectories in prostate cancer survivors.

Investigating the core risk factors of the frailty trajectory holds significant implications for mitigating negative outcomes in older prostate cancer patients. Decision tree modelling has emerged as an effective statistical approach for this purpose.^19^^,^^20^ By visualizing the effects of various variables on outcome indicators through a hierarchical tree diagram, it offers a clear depiction of variable interactions, complementing limitations inherent in traditional regression analysis. Therefore, this study used the decision tree model in conjunction with the logistic regression model as a means of exploring the predictors influencing the frailty trajectories of old patients after radical prostatectomy and identifying the most critical of these factors.

## Methods

### Study design, participants and setting

This longitudinal study was conducted at The First Affiliated Hospital of Wenzhou Medical University in Wenzhou, China, from June 2024 to March 2025. Participants were assessed at four time points, from diagnosis to six months post-surgery. Inclusion criteria were: (1) a pathological diagnosis of prostate cancer and undergoing radical prostatectomy; (2) aged ≥ 60 years; (3) normal language and communication abilities; (4) ability to complete questionnaires and data collection at all follow-up points; (5) awareness of their diagnosis; and (6) given informed consent in this study. The exclusion criteria included: (1) impaired mental status or severe cognitive dysfunction; and (2) prior treatment for other malignant tumours. Sample size estimation was performed using G∗Power software.^21^ With α ​= ​0.05, *β* ​= ​0.10, and an expected effect size of *f* ​= ​0.14, the minimum required sample size was calculated as 92. Allowing for a 30% dropout rate, at least 132 participants were recruited at baseline.

### Data collection

The study commenced with a thorough explanation of its purpose to eligible participants, followed by obtaining informed consent. Data on predictive variables were collected at baseline (T0) through face-to-face interviews conducted by trained investigators before surgery. Frailty assessments were subsequently performed through telephone follow-ups at four postoperative time points: hospital discharge (T1), and 1-, 3-, and 6 months after radical prostatectomy (T2, T3, T4). After each data collection phase, completed questionnaires were reviewed to ensure validity and exclude any incomplete or invalid responses.

### Measurements

#### Sociodemographic and disease characteristics

Age, body mass index (BMI), marital status, living alone, education status, monthly income, and exercise level were surveyed using a self-designed questionnaire. Metastatic status, prostate-specific antigen (PSA) at diagnosis and comorbidity conditions data were obtained from medical records. The comorbidity conditions of patients with prostate cancer was identified according to classifications developed by Charlson.^22^ The comorbidities in this study included: heart attack, congestive heart failure, brain stroke, memory problems, chronic lung illness, connective tissue disease or arthritis, stomach ulcers, liver disease, diabetes, hemiplegic paralysis, and chronic kidney problems.

#### Tilburg Frailty Indicator

Frailty was assessed using the Tilburg Frailty Indicator (TFI), a 15-item tool based on the integral conceptual model of frailty.^23^ It measures three dimensions: physical frailty (8 items), psychological frailty (4 items), and social frailty (3 items), with total scores ranging from 0 to 15. A higher score indicates greater frailty, with a cutoff value of 5. The Chinese version of the TFI demonstrates satisfactory reliability and validity.^24^

#### Geriatric Nutritional Risk Index

Nutritional status was evaluated using the Geriatric Nutritional Risk Index (GNRI), calculated as follows: GNRI ​= ​1.489 ​× ​ALB (g/L) ​+ ​41.7 ​× ​(actual weight / ideal weight). The ideal weight for males was determined as height (cm) - 100 - [(height (cm) - 150) / 4]. When actual weight ​≥ ​ideal weight, the ratio was set to 1. A GNRI score ≤ 98 classified patients into the malnutrition group, while a score > 98 indicated normal nutritional status.^25^

#### Health Literacy Management Scale

Health literacy was assessed using the Health Literacy Management Scale, a 24-item instrument developed by Jordan et al.^26^ It evaluates four dimensions: information acquisition (9 items), communication and interaction (9 items), willingness to improve health (4 items), and willingness to provide financial support (2 items). Responses were scored on a 5-point Likert scale from 1 (“no difficulty at all”) to 5 (“extreme difficulty”), with total scores ranging from 24 to 120; higher scores indicated greater health literacy. The Cronbach's alpha for this study was 0.911.

#### Kessler Psychological Distress Scale

Psychological distress over the past 30 days was measured using the Kessler Psychological Distress Scale (K-10), a 10-item self-report tool.^27^ Each item was rated on a 5-point Likert scale from 1 (“none of the time”) to 5 (“all of the time”), with total scores ranging from 10 to 50. Higher scores reflected greater psychological distress. The Cronbach's alpha for this study was 0.739.

#### Medical Outcome Study Social Support Survey

Social support was evaluated using the Medical Outcome Study Social Support Survey (MOS–SSS–C), a 19-item scale developed by Sherbourn.^28^ It covers four dimensions: tangible support (4 items), emotional and informational support (8 items), positive social interaction (4 items), and affectionate support (3 items). Responses were rated on a 5-point Likert scale from 1 (“never”) to 5 (“always”), with higher scores indicating greater social support. The Cronbach's alpha for this study was 0.912.

### Data analysis

All statistical analyses were conducted using IBM SPSS Statistics version 26.0 and Mplus version 8.3. Descriptive statistics, including means, standard deviations (SD), frequencies, and percentages, were used to summarize the data. Baseline characteristics of participants who completed follow-up and those lost to follow-up were compared using chi-square tests for categorical variables and independent t-tests for continuous variables.

Growth mixture modeling (GMM) was applied to examine frailty trajectories over time and identify distinct subgroups among older patients with prostate cancer post-surgery.^29^ The best-fitting model was guided by combination of several statistical fit indices, including the lowest Information Criteria value (the Akaike Information Criteria (AIC), Bayesian Information Criterion (BIC), and the adjusted BIC (aBIC), entropy values greater than 0.8 and significant *P* values of the Lo-Mendell-Rubin (LMR) Likelihood-ratio test and Bootstrap Likelihood–Ratio Test (BLRT).^30^ The final model was selected based on statistical indicators, clinical interpretability, and class probability (greater than 5% of the sample).^31^

Differences in baseline characteristics and other variables across trajectory groups were analyzed using one-way analysis of variance (ANOVA) and chi-square tests. Variables found to be significant in univariate analyses were included in multivariate logistic regression and a decision tree model to identify predictors of frailty trajectories. The decision tree model was performed using the classification and regression tree (CART). The CART algorithm initiated with a root node, which was recursively split into decision nodes to optimize impurity reduction, as measured by the Gini index. The maximum tree depth was set to three, with a minimum of 50 cases required in parent nodes and 10 in child nodes.^20^ A 10-fold cross-validation approach was applied to fine-tune the CART model. The receiver operating characteristic (ROC) curve analysis was plotted to demonstrate the predictive performance of the CART model for the classification of frailty trajectory. Statistical significance was set at *P* ​< ​0.05.

## Results

### Patient characteristics

Of the 302 patients who met the inclusion criteria, 276 provided written informed consent. However, 28 patients dropped out after baseline, either by withdrawal (*n* ​= ​9) or failure to complete all follow-ups (*n* ​= ​19). Ultimately, 248 patients completed the study. Baseline characteristics of the participants are summarized in Table 1. The mean age was 70.02 ​± ​5.44 years, ranging from 60 to 82 years. A total of 133 participants (53.6%) had a primary school education or lower, and 229 (92.3%) were married. A comparison of baseline characteristics between patients who completed follow-up (*n* ​= ​248) and those who did not (*n* ​= ​28) revealed no statistically significant differences (*P* ​> ​0.05).

**Table 1:** Comparisons of sociodemographic characteristics between patients included in the study and patients lost to follow-up.

Variables	Patients included (*n* ​= ​248)	Patients lost to follow-up (*n* ​= ​28)	*P* value
Age, years, Mean ​± ​SD	70.02 ​± ​5.44	71.71 ​± ​5.28	0.117
BMI, kg/m^2^, Mean ​± ​SD	23.87 ​± ​2.58	24.17 ​± ​3.03	0.621
Marital status, *n* (%)			0.261
Married	229 (92.3)	28 (100.0)	
Divorced or widowed	19 (7.7)	0 (0.0)	
HMIPC (RMB), *n* (%)			0.511
< 3000	111 (44.8)	15 (53.6)	
3000-5000	58 (23.4)	7 (25.0)	
> 5000	79 (31.9)	6 (21.4)	
Education status, *n* (%)			0.706
Primary and below	133 (53.6)	13 (46.4)	
Secondary or high school	95 (38.3)	13 (46.4)	
College and above	20 (8.1)	2 (7.1)	
Live alone, *n* (%)			0.323
No	219 (88.3)	27 (96.4)	
Yes	29 (11.7)	1 (3.6)	
Exercise level, *n* (%)			0.163
≤ 3 time/weeks	125 (50.4)	18 (64.3)	
> 3 time/weeks	123 (49.6)	10 (35.7)	
Smoking history, *n* (%)			0.183
No	164 (66.1)	22 (78.6)	
Yes	84 (33.9)	6 (21.4)	
Metastatic status, *n* (%)			0.752
No	201 (81.0)	22 (78.6)	
Yes	47 (19.0)	6 (21.4)	
Comorbidity, *n* (%)			0.655
< 2	135 (54.4)	14 (50.0)	
≥ 2	113 (45.6)	14 (50.0)	
PSA at diagnosis, M (Q_1_, Q_3_)	9.28 (6.26, 15.67)	10.21 (7.28, 14.18)	0.825
Nutritional status, Mean ​± ​SD	103.46 ​± ​5.23	102.90 ​± ​5.03	0.586
Psychological distress, Mean ​± ​SD	19.80 ​± ​2.69	19.07 ​± ​2.60	0.173
Health literacy, Mean ​± ​SD	85.75 ​± ​10.45	84.71 ​± ​6.68	0.470
Social support, Mean ​± ​SD	65.62 ​± ​9.94	66.11 ​± ​7.26	0.804

BMI, body mass index; HMIPC, household monthly income per capita; PSA, prostate-specific antigen; M, median; SD, standard deviation.

### The prevalence and heterogeneous trajectories of frailty

The prevalence of frailty was highest at hospital discharge, affecting 70.1% of older patients with prostate cancer. This rate declined progressively over time, decreasing to 49.2% at one month, 43.6% at three months, and 39.5% at six months post-surgery. Model fit indices for the trajectory analysis are summarized in Table 2. Among models with different class numbers, the three-class (Class-3) model had the lowest Bayesian Information Criterion (BIC). Additionally, both the Lo-Mendell-Rubin (LMR) test and the Bootstrap-Likelihood Ratio Test (BLRT) were statistically significant for the Class-3 model (*P* ​< ​0.001). Meanwhile, four- or five-class models produced additional categories with small sample sizes that lacked sufficient clinical significance or differentiation, limiting their utility in clinical practice. Considering statistical fit indices and clinical relevance, the three-class quadratic model was identified as optimal, with high average posterior probabilities for group membership (all > 0.9). Thus, the Class-3 model of frailty was identified to be the best-fitting trajectory in the present study.

**Table 2:** Model-Fit indices of the GMM analysis.

Class	AIC	BIC	aBIC	Entropy	LMR(*P*)	BLRT(*P*)	Class probability
1	2837.842	2883.516	2842.306	–	–	–	1
2	2778.599	2838.328	2784.437	0.916	< 0.001	< 0.001	0.561/0.439
3	2736.955	2810.737	2744.166	0.923	< 0.001	< 0.001	0.544/0.145/0.311
4	2740.888	2828.724	2749.473	0.931	0.667	0.452	0.008/0.315/0.133/0.544
5	2735.938	2837.8281	2745.897	0.844	0.322	0.067	0.317/0.163/0.048/0.134/0.338

AIC, Akaike information criterion; BIC, Bayesian Information Criterion; aBIC, adjusted Bayesian Information Criterion; LMR, Lo–Mendell–Rubin likelihood ratio; BLRT, Bootstrapped-Likelihood Ratio Test.

**Fig. 1:**
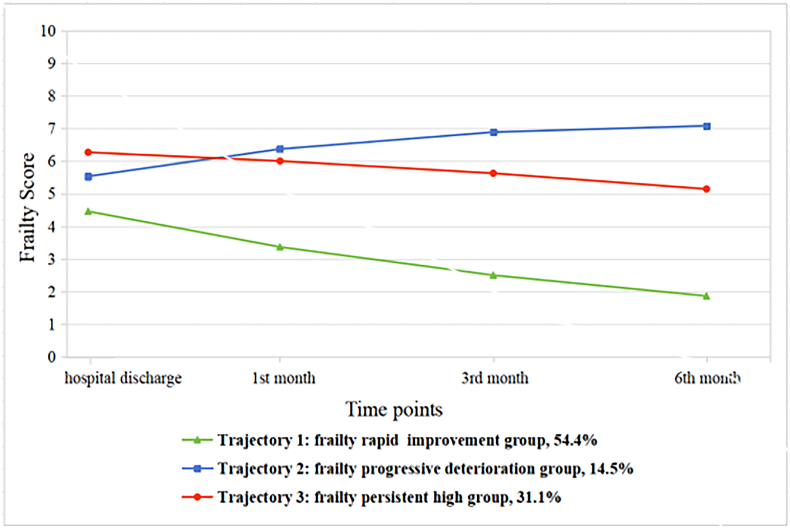
Trajectories of frailty for the 3-class model.

The three frailty trajectories obtained from the model are illustrated in Fig. 1. According to the characteristics of each trajectory, they were classified as: Trajectory 1 (frailty rapid improvement group, 54.4%), Trajectory 2 (frailty progressive deterioration group, 14.5%), and Trajectory 3 (frailty persistent high group, 31.1%). Trajectory 1 had low frailty at baseline, followed by a downward trend over time (*s* ​= ​−1.206, *P* ​< ​0.001). Trajectory 2 had moderate frailty at baseline, with an increasing trend (*s* ​= ​0.997, *P* ​< ​0.001) over time. Trajectory 3 had high frailty at baseline, with no longitudinal change (*s* ​= ​- 0.213, *P ​=* ​0.056).

### Differences in baseline variables

The comparisons of sociodemographic characteristics, clinical factors, and study variables across different frailty trajectories are shown in Table 3. Significant differences were identified among the groups for several factors, including live alone (*χ*^*2*^ ​= ​9.978, *P* ​= ​0.007), exercise level (*χ*^*2*^ ​= ​8.739, *P* ​= ​0.013), metastatic status (*χ*^*2*^ ​= ​9.727, *P* ​= ​0.008), comorbidity (*χ*^*2*^ ​= ​14.444, *P* ​< ​0.001). Additionally, significant differences were also demonstrated in nutritional status (*F* ​= ​25.201, *P* ​< ​0.001), psychological distress (*F* ​= ​54.640, *P* ​< ​0.001), health literacy (*F* ​= ​35.984, *P* ​< ​0.001), social support (*F* ​= ​17.361, *P* ​< ​0.001). Subsequently, these variables were entered into multivariate logistic regression and the decision tree model for further analysis.

**Table 3:** Differences in patients' characteristics and study variables between frailty trajectories among older people with prostate cancer (*N* ​= ​248).

Variables	Trajectory 1 (*n* ​= ​135)	Trajectory 2 (*n* ​= ​36)	Trajectory 3 (*n* ​= ​77)	*χ*^2^*/F*	*P* value
Age, years, Mean ​± ​SD	69.33 ​± ​5.42	71.22 ​± ​4.67	70.65 ​± ​5.68	2.503	0.084
BMI, kg/m^2^, Mean ​± ​SD	24.05 ​± ​2.59	24.23 ​± ​2.51	23.39 ​± ​2.56	2.031	0.133
Marital status, *n* (%)				4.850	0.088
Married	127 (94.1)	30 (83.3)	72 (93.5)		
Divorced or widowed	8 (5.9)	6 (16.7)	5 (6.5)		
HMIPC (RMB), *n* (%)				6.571	0.160
< 3000	51 (37.8)	19 (52.8)	41 (53.2)		
3000-5000	37 (27.4)	8 (22.2)	13 (16.9)		
> 5000	47 (34.8)	9 (25.0)	23 (29.9)		
Education status, *n* (%)				7.036	0.134
Primary and below	66 (48.9)	20 (55.5)	47 (61.0)		
Secondary or high school	53 (39.3)	15 (41.7)	27 (35.1)		
College and above	16 (11.9)	1 (2.8)	3 (3.9)		
Live alone, *n* (%)				9.978	0.007^b^
No	126 (93.3)	27 (75.0)	66 (85.7)		
Yes	9 (6.7)	9 (25.0)	11 (14.3)		
Exercise level, *n* (%)				8.739	0.013^a^
≤ 3 time/weeks	58 (43.0)	25 (69.4)	42 (54.5)		
> 3 time/weeks	77 (57.0)	11 (30.6)	35 (45.5)		
Smoking history, *n* (%)				3.848	0.146
No	90 (66.7)	19 (52.8)	55 (71.4)		
Yes	45 (33.3)	17 (47.2)	22 (28.6)		
Metastatic status, *n* (%)				9.727	0.008^b^
No	119 (88.1)	26 (72.2)	56 (72.7)		
Yes	16 (11.9)	10 (27.8)	21 (27.3)		
Comorbidity, *n* (%)				14.444	< 0.001^b^
< 2	88 (65.2)	13 (36.1)	34 (44.2)		
≥ 2	47 (34.8)	23 (63.9)	43 (55.8)		
PSA at diagnosis, M (Q_1_, Q_3_)	8.54 (6.02, 15.49)	9.64 (6.88, 16.55)	9.55 (7.36, 15.33)	2.937^c^	0.230
Nutritional status, Mean ​± ​SD	105.42 ​± ​4.60	100.62 ​± ​5.34	101.35 ​± ​4.82	25.201	< 0.001^b^
Psychological distress, Mean ​± ​SD	18.56 ​± ​2.22	22.61 ​± ​2.36	20.68 ​± ​2.26	54.640	< 0.001^b^
Health literacy, Mean ​± ​SD	90.19 ​± ​11.11	78.14 ​± ​5.62	81.53 ​± ​6.48	35.984	< 0.001^b^
Social support, Mean ​± ​SD	68.71 ​± ​9.96	59.94 ​± ​7.50	62.87 ​± ​8.98	17.361	< 0.001^b^

BMI, body mass index; HMIPC, household monthly income per capita; PSA, prostate-specific antigen; M, median; SD, standard deviation.

^a^*P* < 0.05.

^b^*P* ​< ​0.01.

^c^Kruskal-waills test.

### Predictors of heterogeneous trajectories of frailty

Table 4 details the results of the multinomial logistic regression analysis, which examined predictors of frailty trajectory membership using Trajectory 1 (frailty rapid improvement group) as the reference. The regression model was significant (χ^2^ ​= ​145.312, *P* ​< ​0.001). Patients with comorbidity ≥ 2 (odds ratio [OR] ​= ​3.319, *P* ​= ​0.019; OR ​= ​2.333, *P* ​= ​0.016) and severe psychological distress (OR ​= ​1.685, *P* ​< ​0.001; OR ​= ​1.211, *P* ​= ​0.027) were more likely to belong to Trajectory 2 and Trajectory 3. Patients who had normal nutritional status (OR ​= ​0.852, *P* ​= ​0.003; OR ​= ​0.864, *P* ​< ​0.001) and great health literacy (OR ​= ​0.900, *P* ​= ​0.006; OR ​= ​0.935, *P* ​= ​0.006) had a higher likelihood of belonging to Trajectory 1.

**Table 4:** Multivariate Logistic regression analysis of predictors for frailty trajectories among older people with prostate cancer (*N* ​= ​248).

Independent variables	Trajectory 2 (*n* ​= ​36)			Trajectory 3 (*n* ​= ​77)		
	OR	95% CI	*P*	OR	95% CI	*P*
Live alone						
No (reference)						
Yes	1.308	0.312–5.484	0.714	1.179	0.369–3.769	0.781
Exercise level						
≤ 3 time/weeks (reference)						
> 3 time/weeks	0.459	0.165–1.272	0.134	0.751	0.375–1.504	0.419
Metastatic status						
No (reference)						
Yes	1.886	0.598–5.950	0.279	1.763	0.736–4.226	0.203
Comorbidity						
< 2 (reference)						
≥ 2	3.319	1.219–9.037	0.019^a^	2.333	1.168–4.662	0.016^a^
Nutritional status	0.852	0.768–0.946	0.003^b^	0.864	0.799–0.935	< 0.001^b^
Psychological distress	1.685	1.314–2.161	< 0.001^b^	1.211	1.023–1.435	0.027^a^
Health literacy	0.900	0.834–0.971	0.006^b^	0.935	0.891–0.981	0.006^b^
Social support	0.953	0.899–1.011	0.107	0.965	0.928–1.003	0.071

OR, odds ratio; CI, confidence interval.

*OR* < 1 indicates a higher likelihood of belonging to Trajectory 1 compared to the reference.

^a^*P* ​< ​0.05.

^*b*^*P* ​< ​0.01.

### Decision tree analysis of heterogeneous trajectories of frailty

**Fig. 2:**
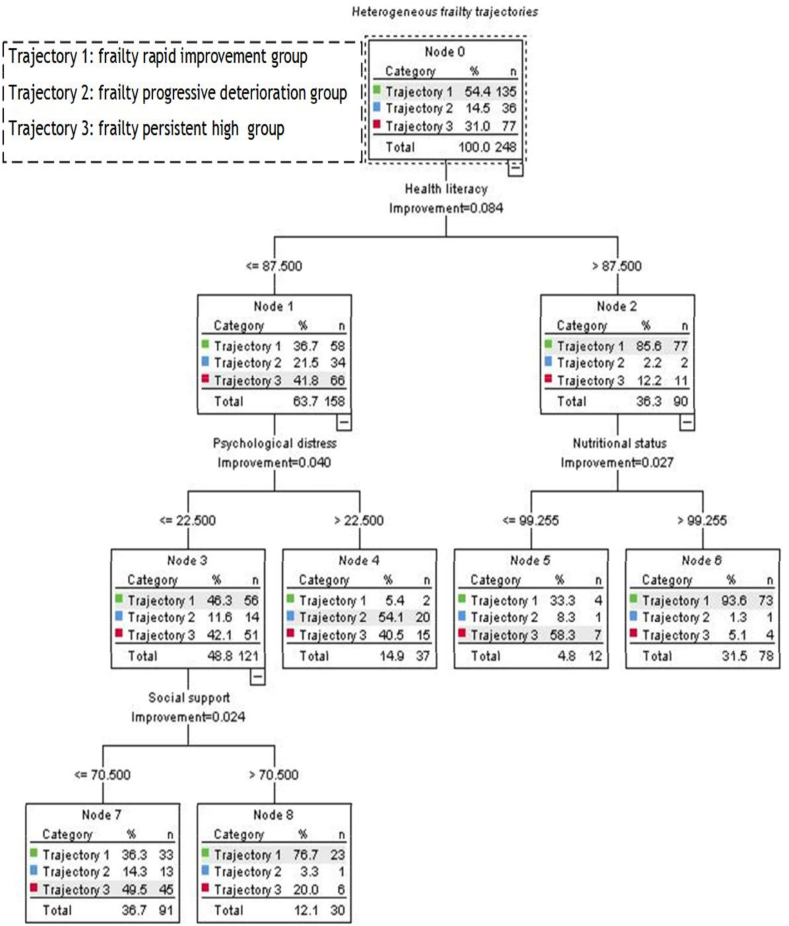
The decision tree model of heterogeneous trajectories of frailty among older people with prostate cancer.

**Fig. 3:**
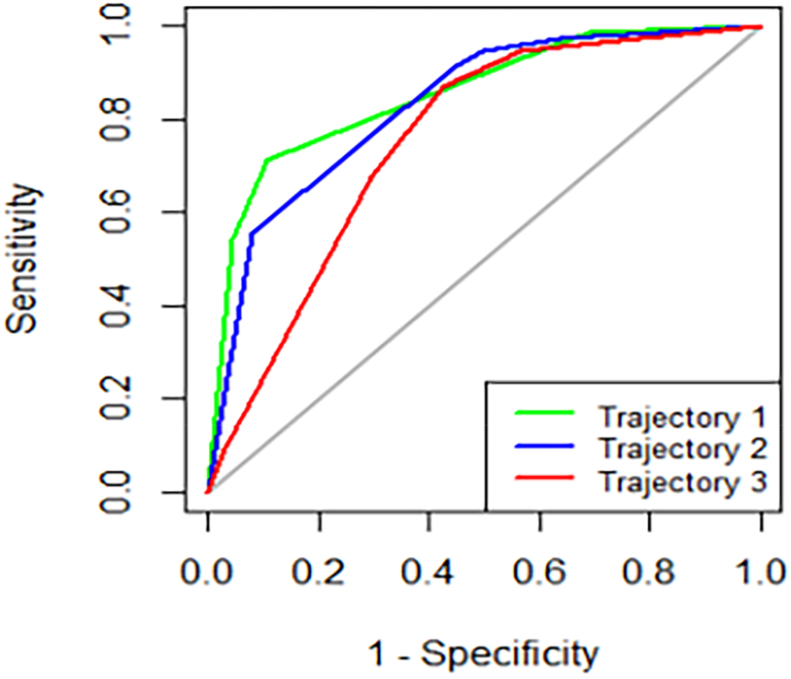
The ROC curve for CART model. ROC, receiver operating characteristic; CART, classification and regression tree.

The decision tree model for heterogeneous frailty trajectories is illustrated in Fig. 2. The model consists of three layers, nine nodes, and five terminal nodes. Decision tree analysis identified health literacy, psychological distress, nutritional status, and social support as main determinants of frailty trajectories in older prostate cancer survivors. Health literacy emerged as the most influential predictor, positioned at the root node. Patients with a health literacy score ≤ 87.5 had a higher likelihood of belonging to Trajectory 3 (41.8%), whereas those with a score > 87.5 were predominantly classified into Trajectory 1 (85.6%). Among those with lower health literacy (≤ 87.5), psychological distress further stratified patients: individuals with a psychological distress score > 22.5 had a greater probability of being in Trajectory 2 (54.1%). In contrast, those with a psychological distress score ≤ 22.5 were further divided based on social support, with patients scoring > 70.5 more likely to be in Trajectory 1. For individuals with health literacy > 87.5, nutritional status provided an additional level of classification. Patients with a nutritional status score > 99.255 had the highest probability of belonging to Trajectory 1 (93.6%), underscoring the protective role of good nutrition in frailty recovery. The ROC curve analysis of the CART model for the classification of frailty trajectory is illustrated in Fig. 3. The area under the curve (AUC), sensitivity, and specificity for CART model were 0.855, 71.1%, 89.4% in the Trajectory 1, 0.831, 55.6%, 92.0% in the Trajectory 2, 0.753, 87.0%, 57.3% in the Trajectory 3.

## Discussion

Based on longitudinal data from a 6-month follow-up study, we aimed to reveal the postoperative progression and heterogeneity of frailty in older patients with prostate cancer. Using Growth Mixture Modeling (GMM), we identified three distinct frailty trajectories: frailty rapid improvement group (54.4%), frailty progressive deterioration group (14.5%), and frailty persistent high group (31.1%). Moreover, both the multivariate logistic regression and decision tree results showed that health literacy, nutritional status, psychological distress, social support and the presence of two or more comorbidities emerged as significant predictors for different trajectories of frailty among older patients with prostate cancer. Our findings offer valuable insights for health care professionals to identify high-risk patients with frailty and early management.

## Frailty heterogeneous trajectories in prostate cancer survivors

In line with previous longitudinal studies, our findings demonstrated that three distinct subgroups of frailty trajectory were identified. The heterogeneity in frailty development concurs with a prior finding on older cancer survivors that frailty change was more a psychological-physiological special effect of cancer through its side effects such as surgery, chemotherapy and negative emotions on all frailty domains during the treatment process.^10^ Consequently, their frailty trajectories fall into three distinct patterns: the “robust”, the “deteriorating” and the “high-risk” group. However, A one-year longitudinal study involving 21,599 older breast cancer survivors identified six trajectory clusters of frailty, including one “low frailty persistence (robust)” and two “frailty transient worsening (resilient)” and three “rapid frailty progression (nonresilient)” trajectory.^32^ These inconsistencies in frailty trajectory classes may be caused by differences in frailty measurement, follow-up time, and patient population features. In this study, three frailty trajectories with high starting points indicate that the degree of frailty among older prostate cancer survivors is relatively severe during the early postoperative period. In this study, three frailty trajectories with high starting points indicate that the degree of frailty among older prostate cancer survivors is relatively severe during the early postoperative period. While the frailty trajectories show a general trend toward improvement in most of prostate cancer survivors, approximately 45% of the patients still experience long-term frailty, with 15% exhibiting deterioration as time progresses. Therefore, health care providers should give due consideration to the features of frailty changes in older patients with prostate cancer across different stages and develop tailored management strategies to reverse the development of heterogeneous frailty trajectories after surgery, thereby enhancing patient-centered care and treatment outcomes.

## Predictors of frailty heterogeneous trajectories

This study found that 6 months after radical prostatectomy, health literacy was the most important factor influencing frailty trajectories among older prostate cancer survivors following radical prostatectomy, as it was at the top level of the decision tree model. Patients with poor health literacy were more likely to fall into the frailty progressive deterioration group and the frailty persistent high group. This finding in line with prior research, which shows that individuals with limited health literacy also had limited understanding and knowledge of health-related matters, contributing to ongoing or worsening frailty.^17^ Health literacy is a core factor in the Information-Motivated Behaviour model, reflecting a patient's capacity to access, process, and comprehend health-related information.^33^ Prostate cancer patients with poor health literacy may be less likely to seek and access supportive resources to manage treatment side effects, such as urinary incontinence or sexual dysfunction.^34^ These challenges can lead to adverse behaviours such as reduced adherence to exercise and rehabilitation treatments, which can hinder physical recovery and thus increase the risk of frailty.^35^ To address these challenges, health care professionals can prioritize supporting older prostate cancer survivors with poor health literacy by using clear, simple language and visual aids, such as pictures or animated videos, to improve their understanding of the disease and its management. Moreover, caregivers should encourage patients to be involved in self-directed frailty management strategies, including maintaining physical activity and seeking support, which may help improve frailty in older patients with prostate cancer.

The present study identified nutritional status as a significant predictor of frailty trajectories among older patients with prostate cancer, based on findings from logistic regression and decision tree modeling. Patients with poor nutritional status increased the likelihood of being in the frailty progressive deterioration group and the frailty persistent high group. The significance of nutritional status in frailty concurs with a previous finding on older gastric cancer survivors that good nutritional status is linked to sustained improvements in frailty during postoperative recovery.^13^ In patients with malnutrition, the organism adapts to this state possibly through several compensatory changes, such as loss of muscle mass and strength and weakened immune function, which heighten frailty risk.^24^ Supporting this, research has shown that a low supply of essential nutrients like energy, protein, and vitamin D are closely linked to frailty among elders.^36^ Notably, nutrition has been identified as a means of slowing the onset and development of frailty in elderly patients and greater adherence to the Mediterranean diet has been shown to have a protective effect against frailty in these patients.^37^ Additionally, combining physical activity with nutritional support such as protein and vitamin D supplements, dietary counselling, and cooking classes has been demonstrated to improve enhance the physical performance, mobility, and overall frailty among elderly patients.^38^ These insights highlight the need for health care professionals to closely monitor the nutritional status of older prostate cancer survivors. For those at risk of malnutrition, timely interventions that integrate nutritional support with physical activity can help delay frailty.

The study's findings from logistic regression and decision tree modelling showed that frailty heterogeneity trajectories were significantly influenced by psychological distress in older prostate cancer survivors after radical prostatectomy. Patients who had higher levels of psychological distress were more likely to fall into the frailty progressive deterioration group and the frailty persistent high group. Similarly, two cross-sectional studies have recently shown a significant positive relationship between severe psychological distress and frailty risk, supporting the results of the current analysis.^39^^,^^40^ The association across psychological distress and frailty may be mediated through multiple pathways. On one hand, psychological distress shares underlying factors in frailty, like chronic inflammation and oxidative stress, which can worsen frailty over time.^16^ Additionally, higher levels of psychological distress often reduce a patient's motivation for rehabilitation, leading to decreased social participation and physical activity.^41^^,^^42^ Lack of exercise can subsequently decrease muscle protein synthesis and metabolism, thereby weakening activity capabilities and increasing vulnerability to frailty.^43^ Furthermore, psychological distress can erode confidence and diminish a sense of purpose, exacerbating fatigue in managing and coping with the disease, which further accelerates frailty progression.^39^ Therefore, health care providers should focus on early screening for psychological distress in older patients with prostate cancer to prevent the development of frailty. For those with severe psychological distress, implementing proven effective strategies like empowerment programs and behavioural activation can support their emotional well-being and encourage active participation in improving their frailty condition.^44^^,^^45^

The decision tree model demonstrated that social support was a predictor of frailty trajectories in older patients with prostate cancer. Patients with greater social support were more likely to be classified in the frailty rapid improvement group. Similarly, a 10-year prospective cohort study involving 466 community-dwelling elderly patients in Japan showed a significant negative correlation between frailty risk and various forms of social support, like emotional and instrumental support.^46^ Social support plays a vital role in mitigating psychological stress by a state of illness in older cancer patients, helping them better manage their illness, adhere to treatment, and improve their frailty status.^47^ For prostate cancer survivors, emotional, familial, and social support can counteract societal stigma related to the disease and associated sexual dysfunction, encouraging social participation and reintegration into social roles after surgery.^48^ Participating in regular group social activities, such as qigong or chess, can also enhance physical mobility and cognitive function, helping to slow the progression of frailty.^49^ Current clinical management guidelines emphasize the importance of tailored social support that addresses the unique needs of frailty patients.^50^ In the future, health care providers should assess both the structural and functional aspects of social support when evaluating frailty risk in older patients with prostate cancer and encourage participation in group activities to slow the progression of frailty.

Moreover, the logistic regression model showed that the presence of two or more comorbidities was linked to frailty trajectories. Specifically, patients with multimorbidity were more likely to experience worsening or persistent frailty after surgery compared to their robust counterparts, aligning with findings from previous studies.^51^ This association can be explained by the common characteristic of multimorbidity and frailty, both of which stem from an accumulation of health deficits over time.^14^ The progression of one condition often exacerbates the other, further increasing the risk of frailty. In clinical practice, targeted preoperative assessment and treatment should be prioritized for older prostate cancer patients with multimorbidity to mitigate frailty progression and improve postoperative outcomes.

Unfortunately, the results do not highlight any prostate cancer-specific factors that may influence frailty trajectories. While factors such as prostate-specific antigen levels and metastatic status are critical for assessing disease severity, their direct association with the development of frailty appears limited. Instead, factors related to postoperative recovery, including complications like lower urinary tract symptoms and subsequent androgen deprivation therapy, may increase the risk of frailty.^52^ Future research focusing on prostate cancer-specific variables, particularly those linked to postoperative treatment and recovery, could offer valuable insights into the unique mechanisms driving frailty in this population.

## Strengths and limitations

To our knowledge, this is the first study to perform a 6-month longitudinal investigation and trajectory analysis of frailty in older patients with prostate cancer after radical prostatectomy. By integrating logistic regression and decision tree modeling, the study provides a systematic method for identifying predictors of frailty trajectories. These findings provide valuable insights for recognizing high-risk patients and informing the development of targeted interventions. However, several limitations should be noted. First, some patients did not complete all follow-up visits and were excluded, which may have resulted in a sample biased toward healthier individuals, potentially influencing model estimates. Second, nearly half of the participants had not recovered from frailty six months postoperatively, highlighting the need for longer follow-up periods to fully understand frailty trajectories over time. Third, the reliability of the k-10 was a bit low, and future research should interpret the findings cautiously, taking cultural and contextual factors into account. Moreover, reliance on self-reported questionnaires for frailty-related measurements may have introduced reporting bias due to inaccurate recall. Finally, the single-center recruitment limits the generalizability of these results in other health care settings and populations. Future research should address these limitations by adopting multi-center designs, extending follow-up durations, and using objective measures alongside self-reports to improve the reliability and applicability of the findings across diverse patient populations.

## Conclusions

The present study identified three distinct frailty trajectories among older patients with prostate cancer 6 months after radical prostatectomy: frailty rapid improvement group, frailty progressive deterioration group, and frailty persistent high group. Patients with low health literacy, poor nutritional status, severe psychological distress, limited social support, or two or more comorbidities were more likely to experience chronic or increased frailty, with health literacy emerging as the most critical predictor. These findings emphasize the need for early, systematic assessment to predict frailty trajectories and guide interventions aimed at mitigating frailty progression. Further future research should develop prediction-oriented interventions targeting the core drivers of frailty development to improve the outcome among older patients with prostate cancer.

## CRediT author contribution statement

**Yongcai Liu:** Conceptualization, Methodology, Writing – original draft. **Jieru Zhou:** Investigation, Data curation, Writing – review & editing. **Yijuan Huang:** Data gathering, Validation. **Xin Yao:** Data gathering, Data curation, Formal analysis. **Xiaoyu Zhang:** Formal analysis. **Jian Cai:** Formal analysis, Investigation. **Haihong Jiang:** Software, Validation. **Wei Chen:** Visualization, Supervision. **Haiyan Li:** Visualization, Supervision, Writing – review & editing. All authors have read and approved the final manuscript.

## Ethics statement

This study was approved by the Medical Ethical Committee of The First Affiliated Hospital of Wenzhou Medical University (Approval No. KY2024-144) and was conducted in accordance with the 1964 Helsinki Declaration and its later amendments or comparable ethical standards. Written Informed consent was obtained before patients' participation.

## Data availability statement

The data that support the findings of this study are available from the corresponding author, HL, upon reasonable request.

## Declaration of generative AI and AI-assisted technologies in the writing process

No AI tools/services were used during the preparation of this work.

## Funding

This work was supported by Scientific Research Fund of Zhejiang Provincial Education Department (Grant No. Y202457322); Zhejiang Provincial Medical and Health Science and Technology Project (Grant No. 2025KY101; 2024KY141); Discipline Cluster of Oncology, Wenzhou Medical University, China (Grant No. z1-2023003). The funders had no role in considering the study design or in the collection, analysis, interpretation of data, writing of the report, or decision to submit the article for publication.

## Declaration of competing interest

The authors declared that there have no conflicts of interest to this work.
